# The database of eye-movement measures on words in Chinese reading

**DOI:** 10.1038/s41597-022-01464-6

**Published:** 2022-07-15

**Authors:** Guangyao Zhang, Panpan Yao, Guojie Ma, Jingwen Wang, Junyi Zhou, Linjieqiong Huang, Pingping Xu, Lijing Chen, Songlin Chen, Junjuan Gu, Wei Wei, Xi Cheng, Huimin Hua, Pingping Liu, Ya Lou, Wei Shen, Yaqian Bao, Jiayu Liu, Nan Lin, Xingshan Li

**Affiliations:** 1 CAS Key Laboratory of Behavioral Science, Institute of Psychology, Beijing, China; 2 Department of Psychology, University of Chinese Academy of Sciences, Beijing, China; 3 School of Psychology, Beijing Language and Culture University, Beijing, China

**Keywords:** Human behaviour, Human behaviour

## Abstract

Eye movements are one of the most fundamental behaviors during reading. A growing number of Chinese reading studies have used eye-tracking techniques in the last two decades. The accumulated data provide a rich resource that can reflect the complex cognitive mechanisms underlying Chinese reading. This article reports a database of eye-movement measures of words during Chinese sentence reading. The database contains nine eye-movement measures of 8,551 Chinese words obtained from 1,718 participants across 57 Chinese sentence reading experiments. All data were collected in the same experimental environment and from homogenous participants, using the same protocols and parameters. This database enables researchers to test their theoretical or computational hypotheses concerning Chinese reading efficiently using a large number of words. The database can also indicate the processing difficulty of Chinese words during text reading, thus providing a way to control or manipulate the difficulty level of Chinese texts.

## Abstract (summary)

**Table:** 

Measurement(s)	eye movement
Technology Type(s)	eye tracking
Factor Type(s)	word frequency • word length
Sample Characteristic - Organism	Human
Sample Characteristic - Environment	laboratory environment

## Background & Summary

Skilled readers move their eyes rapidly through text, approximately four to five times per second, and can achieve a reading speed of approximately 250 words per minute^1,2^. When and where the eyes move are influenced by cognitive processes during reading; thus, eye movements provide rich information for studying the underlying cognitive mechanisms of reading^3,4^. Eye movements have been used extensively to study the cognitive mechanisms of alphabetic reading, particularly in English. A growing number of studies have used eye-tracking techniques to study Chinese reading in the last two decades. These studies have found many similarities between Chinese and alphabetic reading. For example, the fixation time and fixated probability on Chinese and alphabetic words are modulated by word frequency and word length^3,5^. Additionally, the script-specific mechanisms of Chinese reading, such as how Chinese readers segment words and program their eye movements without the aid of inter-word spaces, have been studied extensively^6,7^.

Traditional factor-designed experiments have been fruitful in revealing cognitive mechanisms in Chinese reading. However, a large-scale eye movement database can provide valuable information not available in small-scale experimental studies. Multiple complex variables affect eye movements during reading and it is challenging to manipulate or control all of them simultaneously in controlled experiments. It is also often questioned whether conclusions based on dozens of words or sentences can be generalized to unexamined linguistic materials^8^. A large-scale eye-movement database can overcome these problems, allowing researchers to simultaneously examine the effects of multiple factors on reading behaviors and ensure the generalizability of the conclusions. Furthermore, researchers can generate and examine new hypotheses using big data, making data usage wider than the original experiments.

Several eye-tracking databases of alphabetic reading have been established, such as the Potsdam corpus^9,10^, the Provo corpus^11^, and the Ghent Eye Movement Corpus (GECO)^12^. These databases have been used in many aspects of reading research, such as examining the impacts of linguistic and other variables on text reading^9,10^, improving the computational models for alphabetic text reading^13,14^, and investigating the relationship between first- and second-language reading^15–17^. Recently, corpus analysis has also been used to investigate the mechanisms of Chinese reading^5,18,19^. However, the existing eye-tracking databases of Chinese reading are relatively small. A larger database is strongly needed, which can be used to investigate the complex cognitive mechanisms underlying Chinese reading and can be more easily compared with eye-tracking databases of alphabetic reading to reveal the similarity and difference between Chinese and alphabetic reading^20^.

**Fig. 1:**
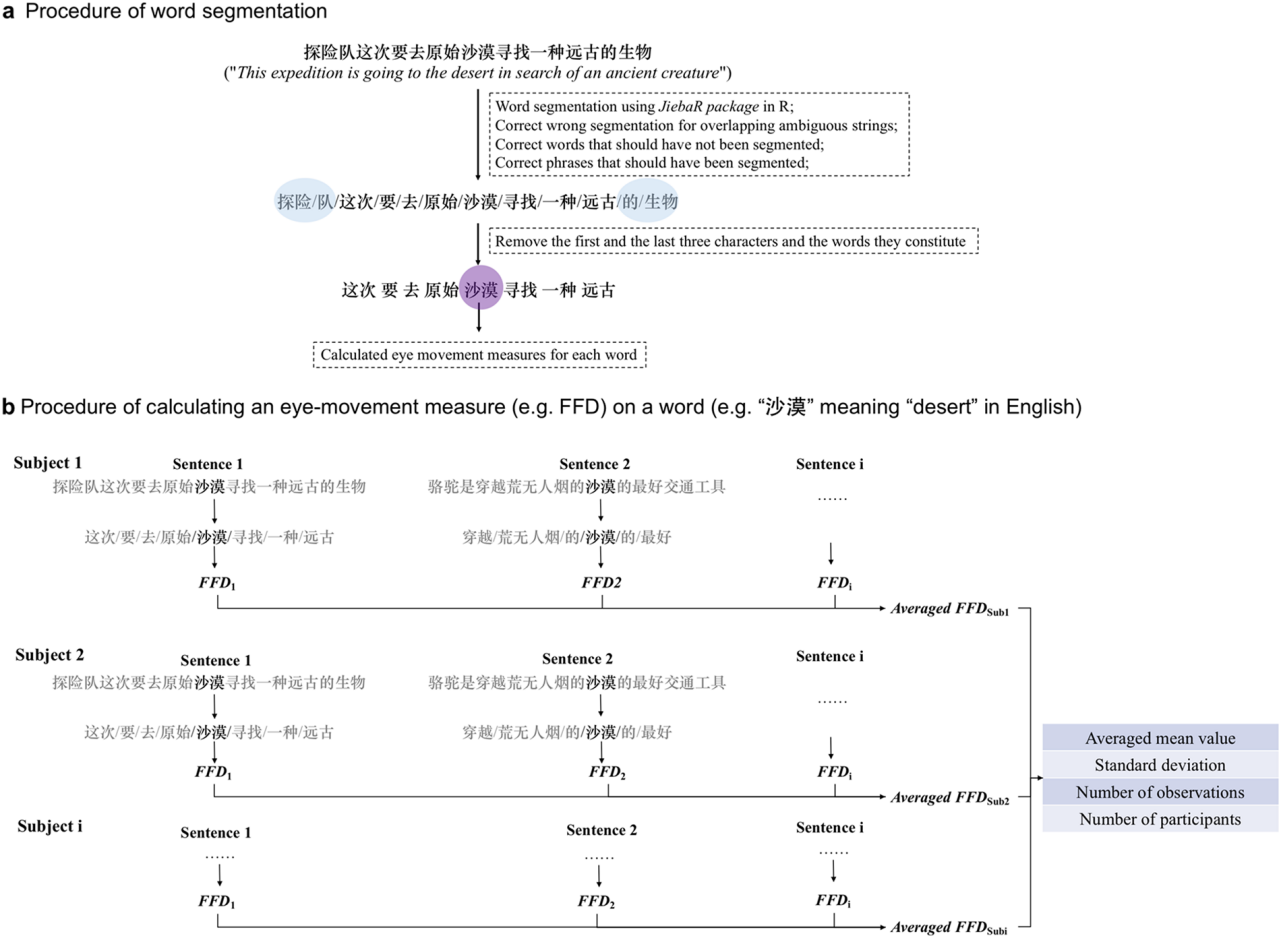
Schematic visualization of word segmentation and measure calculation. *Note*. Panel (**a**) shows the procedure of word segmentation for one sentence. Panel (**b**) shows an example of the procedure of calculating an eye-movement measure (i.e., FFD) on a word (e.g., “沙漠” meaning “desert” in English).

Here we report a sizeable eye-tracking database, the Chinese Eye-Movement Database, which summarizes nine eye-movement measures for over 8,000 different Chinese words. Our database was based on data collected from 57 eye-movement experiments using a sentence-reading task and totally 1,718 participants. Figure 1 presents a schematic of the procedure used to construct the database.

## Methods

### Data acquisition

Data were obtained from 1,718 participants across 57 experiments. All experiments were approved by and performed in accordance with guidelines and regulations of the Institutional Ethics Committee at the Institute of Psychology of the Chinese Academy of Sciences. All the participants were college students and native Chinese speakers with normal or corrected-to-normal vision. Each participant read and signed the informed consent form before the experiment. In all experiments, native Chinese readers silently read sentences naturally for comprehension, with no special experimental paradigm (e.g., the moving window paradigm or gaze-contingent boundary paradigm) adopted. The eye-tracker was calibrated for each participant during each experiment before the task. The materials were presented on a 21-inch CRT monitor (Sony G520; resolution: 1,024 × 768 pixels) connected to a Dell PC. Participants viewed the stimuli approximately 58 cm away from the monitor. They placed their chin on a chin rest to minimize head movements and read sentences binocularly while only their right eyes were monitored. Eye movements were recorded using an EyeLink 1000 eye-tracking system with a sampling rate of 1,000 Hz.

The materials used in all experiments included 8,015 different natural Chinese sentences. Sentences shorter than 15 characters were excluded. After this, 7,577 sentences remained, with each containing 15–35 characters (mean 22.48). The sentences were all of a high semantic plausibility (i.e., the rating scores were higher than 4.5 on a 7-point scale, where higher scores indicate higher plausibility). This was based on the assessment of the participants who did not participate in the eye-tracking experiments.

### Word segmentation

The word segmentation procedure is shown in Fig. 1a. Because there are no explicit markers to demarcate words in Chinese text, we used a package called *jiebaR*^21^ in *R*^22^ to segment words. Segmentation was performed primarily based on the *Lexicon of Common Words in Contemporary Chinese (Draft)*^23^. Words not included in this dictionary were segmented based on the default dictionary in *jiebaR*^21^. Afterward, the words were manually checked to correct segmentation errors, particularly in the following three situations. First, overlapping ambiguous strings (OASs) may have been incorrectly segmented. An OAS is a string of characters (e.g., “学生活,” herein referred to as characters A, B, and C, respectively), wherein the middle character can form distinct words with the characters on both its left (e.g., word “学生,” meaning “student” in English) and right (e.g., word “生活,” meaning “life” in English)^24–28^. In some situations, the software incorrectly segments AB-C as A-BC or segments A-BC as AB-C. Second, the word may have been segmented incorrectly into several words. For example, “马上” (meaning “immediately”) was incorrectly segmented into two one-character words (i.e., “马,” meaning “horse,” and “上,” meaning “up”). In this case, they are adjusted to a single word. Third, phrases may have been treated incorrectly as whole words. For example, a noun–noun phrase, such as “英语文学” (meaning “English literature”) should be segmented into two words, “英语” (meaning “English”) and “文学” (meaning “literature”), which was instead identified as one word.

### Pre-processing and calculation of eye-movement measures

The eye-movement data were pre-processed using the *EyeDoctor 0.6.5* software developed by *UMASS Eye-Tracking Lab*. Sentences in which participants made more than three blinks while reading were excluded from the analyses, as were fixations and saccades that contained blinks. Furthermore, fixations longer than 1,000 ms or shorter than 80 ms were excluded.

Eye-movement measures for each word were calculated using the *DPEEM* package^29^ in *R*^22^. Considering that readers do not always start reading from the first character of a sentence and there are more blinks at the beginning, the first three characters were excluded from the analyses. Moreover, the last three characters in a sentence were excluded from the subsequent analyses to avoid the wrap-up effect^30^. Words containing any excluded character from the analyses were eliminated. Additionally, the words not listed in the *Lexicon of Common Words in Contemporary Chinese (Draft)*^23^ were excluded from the analyses. In total, 8,551 different words were included, including 1,354 one-character words, 6,128 two-character words, 547 three-character words, and 522 four-character words. We calculated nine eye-movement measures for each word. Table 1 presents the definitions and abbreviations of these measures. As shown in Fig. 1b, for each measure of the given word, we first calculated the mean values of each participant. The average mean values and corresponding standard deviations were then calculated across participants. Table 2 shows the descriptive information of the nine measures on words of different length.

**Table 1:** Definitions and Abbreviations of the Nine Eye-Movement Measures.

Eye-Movement Measures	Abbreviations	Definition
First fixation duration*	FFD	Duration of the first fixation on the target word
Gaze duration*	GD	Sum of the fixation durations before the target word is exited to the right or left during first-pass reading
First-pass reading fixated proportion*	FPF	Proportion that the target word is fixated during the first-pass reading
Fixation number^+^	FN	Total number of fixations on the target word
Proportion regression in^+^	RI	Proportion of regression into the target word
Proportion regression out^+^	RO	Proportion of regression out from the target word
Saccade length toward the target from the left^+^	LI_left	Length of saccade into the target word when the word is first fixated from the left side (unit: character)
Saccade length from the target to the right^+^	LO_right	Length of the saccade from target word to the right after the word first fixated (unit: character)
Total fixation duration^+^	TT	Sum of the fixation durations on the target word

*Note*. *Main measures in the database. ^+^Supplementary measures in the database.

**Table 2:** Mean Value (Standard Deviation) of the Eye-Movement Measures on Words of Different Length.

	Word length (number of characters)			
	1	2	3	4
Sample size	1354	6128	547	522
FFD (ms)	264 (38)	264 (33)	259 (30)	254 (32)
GD (ms)	270 (43)	307 (59)	364 (90)	414 (106)
FPF	0.459 (0.132)	0.783 (0.123)	0.927 (0.082)	0.963 (0.052)
FN	0.747 (0.255)	1.460 (0.470)	1.928 (0.582)	2.304 (0.654)
RI	0.145 (0.100)	0.223 (0.127)	0.232 (0.132)	0.208 (0.135)
RO	0.146 (0.102)	0.242 (0.137)	0.262 (0.158)	0.298 (0.184)
LI_left (characters)	2.700 (2.761)	2.801 (1.650)	2.941 (1.195)	3.142 (1.103)
LO_right (characters)	3.338 (7.839)	3.450 (5.318)	3.390 (5.861)	3.935 (4.93)
TT (ms)	338 (86)	438 (139)	509 (178)	573 (190)

## Data Records

The database is freely available on OSF repository^31^ under the CC BY 4.0 License. The raw data are provided in the file “Raw Data.txt”, “Sentences.xlsx” and “ROIs.xlsx”.

The descriptive statistics of the eye-movement measures of each of the 8,551 different words are provided in the files named “MainMeasures.xlsx” and “Supplementary Measures.xlsx”). “Main Measures.xlsx” file contains information regarding first fixation duration (FFD), gaze duration (GD), and first-pass reading fixated proportion (FPF), while the “Supplementary Measures.xlsx” file contains information regarding the remaining six measures (for definitions, see Table 1). The following information is available in each file:

1. The column named “words” provides the words for which the eye-movement measures were calculated, e.g., “钱” (meaning “Money” in English).
2. The columns starting with “Mean_” provide the mean values of the eye-movement measures, e.g., the column named “Mean_FFD” provides the mean value of FFD for each word.
3. The columns starting with “SD_” provide the standard deviations (SDs) of the eye-movement measures, e.g., the column named “SD_FFD” provides the SD of FFD for each word.
4. The columns starting with “Numobs_” provide the number of observations of each word on each eye-movement measure, e.g., the column named “Numobs_FFD” provides the number of observations of each word on FFD.
5. The columns started with “Numsub_” provide the number of participants that the eye-movement measures were calculated based on, e.g., the column named “Numsub_FFD” provides the number of participants that the FFDs were calculated based on.
6. The column named “num_sentence” provides the number of sentences that contain each word.
7. The column named “frequency_subtle_based” provides subtitle-based word frequency of the corresponding word^32^.

### Structure of the Raw Data

All raw data are available on the website 10.17605/OSF.IO/94WUE. All sentences and their specific sequence labels (indicated by column named “Sentence_ID”) are available in the file named “Sentence.xlsx”. The file named “Raw Data.txt” contains all raw data. In this file, each row provides information for one fixation observed by a subject during reading. The seven columns provide the following information.

1. The column named “Experiment” shows which experiment the fixation belongs to.
2. The column named “Subject” shows which participant the fixation was observed from.
3. The column named “Sentence_ID” shows which sentence the fixation was observed while reading, which can be used to find the corresponding sentence in “Sentences.xlsx” file.
4. The column named “X_Position” shows the horizontal coordinates of the fixation as measured by characters. The position of the first character of a line is encoded as zero. Fixations that fall outside the scope of sentences are invalid, and their horizontal coordinates are encoded as “−1”. These fixations were not used to calculate eye-movement measures.
5. The column named “Y_Position” shows the vertical coordinates of the fixation as measured by lines of text. Because all sentences were presented within a single line, vertical coordinates of all fixations within the scope of sentences are zero. For fixations out of the scope of sentences, vertical coordinates are encoded as “−1”.
6. The column named “Onset_Time” shows the onset of one fixation (unit: ms).
7. The column named “Offset_Time” shows the offset of one fixation (unit: ms). Fixation duration can be calculated from subtracting “onset” from “offset”.

“ROIs.xlsx” file contains information of words in sentences for each experiment. This information was used in calculating eye-movement measures. The six columns provide the following information.

1. The column named “Experiment” shows which experiment the word belongs to.
2. The column named “Sentence_ID” shows which sentence the word belongs to, which can be used to find the corresponding sentence in “Sentences.xlsx” file.
3. The column named “ROI_Beginning” shows the horizontal coordinates of the first character of the word in the current sentence.
4. The column named “Word_Length” shows the word length.
5. The column named “Word_Order” indicates order of the word in the current sentence.
6. The column named “Words” shows the current word.

## Technical Validation

### Qualitative validation

The following criteria assured the data quality of the present database. First, all data were collected in the same laboratory using the same protocols and tasks (i.e., silent sentence reading). Second, the participants recruited in the experiments were all college students and native Chinese speakers with normal or corrected-to-normal vision. Third, eye-movement measures were calculated using the previously validated analysis procedure. Together, these homogeneities minimize the variation of the experimental environment, tasks, procedures, and participants.

### Quantitative validation

To quantitatively validate the database, we analyzed the impacts of word frequency and word length on three primary measures—FFD, GD, and FPF to examine whether the classic findings of small-scale experimental eye-tracking studies can be replicated using our database. These effects are well demonstrated^3,5^ and have often been used to validate computational models for reading^33,34^. We examined the effects in the current database by fitting a general linear model for each measure with log-transformed word frequency and word length as predictors. Word frequency was obtained from SUBTLEX-CH^32^, and was treated as a continuous variable, and word length was treated as a factor variable, with successive differences coding adopted. As shown in Table 3, the word frequency and word length effects were replicated in the current database. Words with higher frequency received shorter FFD, shorter GD, and lower FPF. The longer words received shorter FFD, longer GD, and higher FPF.

**Table 3:** Results for the Effects of Word Frequency and Word Length on the Main Eye-Movement Measures.

Dependent variables	Independent variables	*b* value	Cohen’s *d*	*t* value
FFD	Log-transformed word frequency	−11.170	−0.253	−21.026^***^
	2-char words vs 1-char words	−7.568	−0.230	−7.328^***^
	3-char words vs 2-char words	−11.626	−0.353	−7.499^***^
	4-char words vs 3-char words	−6.824	−0.207	−3.154^**^
GD	Log-transformed word frequency	−22.350	−0.249	−23.031^***^
	2-char words vs 1-char words	19.533	0.291	10.354^***^
	3-char words vs 2-char words	40.469	0.603	14.291^***^
	4-char words vs 3-char words	46.267	0.690	11.708^***^
FPF	Log-transformed word frequency	−0.045	−0.183	−23.224^***^
	2-char words vs 1-char words	0.291	1.599	78.196^***^
	3-char words vs 2-char words	0.127	0.697	22.685^***^
	4-char words vs 3-char words	0.024	0.133	3.093^**^

*Note*. *p < 0.05, **p < 0.01, ***p < 0.001. Abbreviations: FFD, first fixation duration; GD, gaze duration; FPF, first-pass reading fixation proportion.

Considering that the number of observations of a word may substantially impact the data reliability of it, we re-conducted the analyses above by dividing the words into quarters based on the number of observations for each measure. Table 4 shows the lexical information for each quarter, and Supplementary Table 1 shows the results. There were expected word frequency and word length effects in each quarter, even in quarters where words had fewer observations (i.e., Quarter 1 and Quarter 2).

**Table 4:** Lexical Information of the Four Quarters of Words Divided Based on the Number of Observations.

Measures	Quarters	Log-transformed word frequency		Number of observations		Sample size in different word length (unit: character)			
		Mean (SD)	Range	Mean (SD)	Range	1	2	3	4
FFD	Quarter 1	0.632 (0.541)	[0.013, 3.425]	8 (3)	[1, 13]	450	1250	172	136
	Quarter 2	0.689 (0.554)	[0.013, 3.197]	20 (4)	[13, 26]	263	1443	126	176
	Quarter 3	0.864 (0.621)	[0.013, 4.598]	37 (9)	[26, 55]	242	1567	121	78
	Quarter 4	1.598 (0.809)	[0.013, 4.700]	219 (892)	[55, 35299]	396	1542	49	22
GD	Quarter 1	0.632 (0.541)	[0.013, 3.425]	8 (3)	[1, 13]	450	1250	172	136
	Quarter 2	0.689 (0.554)	[0.013, 3.197]	20 (4)	[13, 26]	263	1443	126	176
	Quarter 3	0.864 (0.621)	[0.013, 4.598]	37 (9)	[26, 55]	242	1567	121	78
	Quarter 4	1.598 (0.809)	[0.013, 4.700]	219 (892)	[55, 35299]	396	1542	49	22
FPF	Quarter 1	0.572 (0.517)	[0.013, 3.425]	10 (3)	[2, 15]	362	1268	230	148
	Quarter 2	0.666 (0.524)	[0.013, 3.197]	23 (5)	[15, 30]	190	1544	87	187
	Quarter 3	0.905 (0.598)	[0.013, 3.527]	46 (11)	[30, 70]	301	1534	113	60
	Quarter 4	1.639 (0.797)	[0.013, 4.700]	355 (2073)	[70, 83658]	498	1456	38	17

*Note*. Quarters of each measure were divided based on the number of observations of words in ascending order, with each quarter containing 2008-2009 words. Abbreviations: FFD, first fixation duration; GD, gaze duration; FPF, first-pass reading fixation proportion; SD, standard deviation.

In addition to the subtitle-based word frequency, we also used the word frequency from the Chinese Linguistic Data Consortium (2003) corpus to perform the same analyses above. The results are shown in Tables 5, 6 and Supplementary Table 2, which is similar to those using frequency from SUBTLEX-CH^32^ and thus also validated the current database.

**Table 5:** Results for the Effects of Word Frequency and Word Length on the Main Eye-Movement Measures.

Dependent variables	Independent variables	*b* value	Cohen’s *d*	*t* value
FFD	Log-transformed word frequency	−10.526	−0.249	−17.607^***^
	2-char words vs 1-char words	−11.349	−0.346	−9.083^***^
	3-char words vs 2-char words	−10.388	−0.317	−7.115^***^
	4-char words vs 3-char words	−7.453	−0.227	−3.772^***^
GD	Log-transformed word frequency	−25.394	−0.276	−22.154^***^
	2-char words vs 1-char words	6.727	0.094	2.808^**^
	3-char words vs 2-char words	45.76	0.64	16.347^***^
	4-char words vs 3-char words	42.77	0.598	11.289^***^
FPF	Log-transformed word frequency	−0.054	−0.235	−25.514^***^
	2-char words vs 1-char words	0.259	1.441	58.157^***^
	3-char words vs 2-char words	0.12	0.669	23.113^***^
	4-char words vs 3-char words	0.021	0.116	2.949^**^

*Note*. *p < 0.05, **p < 0.01, ***p < 0.001. Abbreviations: FFD, first fixation duration; GD, gaze duration; FPF, first-pass reading fixation proportion.

**Table 6:** Lexical Information of the Four Quarters of Words Divided Based on the Number of Observations.

Measures	Quarters	Log-transformed word frequency		Number of observations		Sample size in different word length (unit: character)			
		Mean (SD)	Range	Mean (SD)	Range	1	2	3	4
FFD	Quarter 1	0.792 (0.659)	[0.009, 3.386]	8 (3)	[1, 13]	342	1376	212	171
	Quarter 2	0.842 (0.641)	[0.017, 3.312]	19 (4)	[13, 26]	248	1470	150	233
	Quarter 3	1.024 (0.661)	[0.023, 3.347]	36 (8)	[26, 53]	234	1653	126	88
	Quarter 4	1.697 (0.778)	[0.016, 4.46]	212 (872)	[53, 35299]	406	1613	56	26
GD	Quarter 1	0.792 (0.659)	[0.009, 3.386]	8 (3)	[1, 13]	342	1376	212	171
	Quarter 2	0.842 (0.641)	[0.017, 3.312]	19 (4)	[13, 26]	248	1470	150	233
	Quarter 3	1.024 (0.661)	[0.023, 3.347]	36 (8)	[26, 53]	234	1653	126	88
	Quarter 4	1.697 (0.778)	[0.016, 4.46]	212 (872)	[53, 35299]	406	1613	56	26
FPF	Quarter 1	0.727 (0.627)	[0.009, 3.386]	10 (3)	[2, 15]	278	1358	283	182
	Quarter 2	0.799 (0.584)	[0.017, 3.199]	22 (5)	[15, 30]	169	1593	98	241
	Quarter 3	1.064 (0.649)	[0.023, 3.326]	44 (11)	[30, 67]	272	1629	124	76
	Quarter 4	1.765 (0.763)	[0.016, 4.46]	343 (2028)	[67, 83658]	511	1532	39	19

*Note*. Quarters of each measure were divided based on the number of observations of words in ascending order, with each quarter containing 2101 words. Abbreviations: FFD, first fixation duration; GD, gaze duration; FPF, first-pass reading fixation proportion; SD, standard deviation.

## Usage Notes

The current database is available at OSF repository^31^. This database can contribute to understanding the cognitive mechanisms underlying Chinese reading in several ways. First, the current database can be analyzed to test new theoretical hypotheses regarding Chinese reading. Second, it can be used to find the optimal parameters for new computational models of Chinese reading and can provide benchmark data to evaluate them. Third, the current database, combined with the existing eye-tracking databases of alphabetic reading, can be used to investigate the mechanisms of reading cross-linguistically^20^. Finally, the large-scale eye-movement measures reported in the database can serve as indicators of word-processing difficulty in Chinese text reading. Thus, it can be used to control or manipulate the difficulty level of reading stimuli, which is valuable in scientific research and potentially helpful for selecting suitable reading materials for readers with different literacy skills.

## Supplementary information

Supplementary Table 1

Supplementary Table 2

## Data Availability

The codes for eye-movement measure calculating, descriptive statistics and quantitative validation are available on OSF repository^31^. There were two R script files. The file named “Main.R” contained the R codes for data calculation and validation, and all of the functions used are contained in the file named “functions.R”.
