# Supplementary material for: The database of eye-movement measures on words in Chinese reading
Source: Sci Data. 2022 Jul 15;9:411. doi: 10.1038/s41597-022-01464-6 (PMC9287311; doi:10.1038/s41597-022-01464-6)
Supplement: Supplementary file 1 — Supplementary Table 1 [file 41597_2022_1464_MOESM1_ESM.docx]

**Supplementary Table 1**

*Results for the Effects of Word Frequency and Word Length on the Main Eye-Movement Measures in Four Quarters of Words Divided Based on the Number of Observations*

| Dependent  variables | Independent variables | Quarters | *b* value | Cohen’s *d* | *t* value |
| --- | --- | --- | --- | --- | --- |
| FFD | Log-transformed word frequency | Quarter 1 | -12.258 | -0.144 | -5.815^***^ |
|  |  | Quarter 2 | -11.656 | -0.194 | -7.846^***^ |
|  |  | Quarter 3 | -8.849 | -0.211 | -8.486^***^ |
|  |  | Quarter 4 | -6.854 | -0.322 | -13.101^***^ |
|  | 2-char words vs 1-char words | Quarter 1 | -1.464 | -0.032 | -0.543 |
|  |  | Quarter 2 | -5.417 | -0.163 | -2.301^*^ |
|  |  | Quarter 3 | -7.920 | -0.304 | -4.075^***^ |
|  |  | Quarter 4 | -9.049 | -0.526 | -8.648^***^ |
|  | 3-char words vs 2-char words | Quarter 1 | -18.705 | -0.405 | -5.025^***^ |
|  |  | Quarter 2 | -7.305 | -0.220 | -2.398^*^ |
|  |  | Quarter 3 | -12.002 | -0.461 | -4.972^***^ |
|  |  | Quarter 4 | -9.203 | -0.535 | -3.842^***^ |
|  | 4-char words vs 3-char words | Quarter 1 | -1.940 | -0.042 | -0.371 |
|  |  | Quarter 2 | -14.947 | -0.449 | -3.940^***^ |
|  |  | Quarter 3 | -2.874 | -0.110 | -0.774 |
|  |  | Quarter 4 | -7.703 | -0.448 | -1.822 |
| GD | Log-transformed word frequency | Quarter 1 | -25.906 | -0.158 | -7.055^***^ |
|  |  | Quarter 2 | -25.917 | -0.200 | -9.018^***^ |
|  |  | Quarter 3 | -21.229 | -0.242 | -10.769^***^ |
|  |  | Quarter 4 | -11.761 | -0.296 | -13.473^***^ |
|  | 2-char words vs 1-char words | Quarter 1 | 30.965 | 0.349 | 6.587^***^ |
|  |  | Quarter 2 | 27.361 | 0.382 | 6.007^***^ |
|  |  | Quarter 3 | 17.514 | 0.322 | 4.767^***^ |
|  |  | Quarter 4 | 11.988 | 0.373 | 6.866^***^ |
|  | 3-char words vs 2-char words | Quarter 1 | 23.461 | 0.264 | 3.618^***^ |
|  |  | Quarter 2 | 58.341 | 0.814 | 9.899^***^ |
|  |  | Quarter 3 | 36.740 | 0.675 | 8.050^***^ |
|  |  | Quarter 4 | 38.182 | 1.187 | 9.553^***^ |
|  | 4-char words vs 3-char words | Quarter 1 | 86.670 | 0.976 | 9.512^***^ |
|  |  | Quarter 2 | 9.714 | 0.136 | 1.323 |
|  |  | Quarter 3 | 34.504 | 0.634 | 4.913^***^ |
|  |  | Quarter 4 | 30.967 | 0.963 | 4.389^***^ |
| FPF | Log-transformed word frequency | Quarter 1 | -0.047 | -0.118 | -6.627 |
|  |  | Quarter 2 | -0.032 | -0.105 | -6.108^***^ |
|  |  | Quarter 3 | -0.024 | -0.087 | -5.506^***^ |
|  |  | Quarter 4 | -0.017 | -0.087 | -7.359^***^ |
|  | 2-char words vs 1-char words | Quarter 1 | 0.295 | 1.420 | 31.090^***^ |
|  |  | Quarter 2 | 0.312 | 1.961 | 34.744^***^ |
|  |  | Quarter 3 | 0.297 | 1.816 | 41.924^***^ |
|  |  | Quarter 4 | 0.297 | 1.868 | 69.396^***^ |
|  | 3-char words vs 2-char words | Quarter 1 | 0.110 | 0.527 | 10.155^***^ |
|  |  | Quarter 2 | 0.112 | 0.703 | 9.079^***^ |
|  |  | Quarter 3 | 0.136 | 0.834 | 13.201^***^ |
|  |  | Quarter 4 | 0.157 | 0.990 | 12.784^***^ |
|  | 4-char words vs 3-char words | Quarter 1 | 0.028 | 0.135 | 1.776 |
|  |  | Quarter 2 | 0.022 | 0.137 | 1.504 |
|  |  | Quarter 3 | 0.031 | 0.187 | 1.808 |
|  |  | Quarter 4 | 0.033 | 0.205 | 1.490 |

*Note.* Quarters of each measure were divided based on the number of observations of words in ascending order, with each quarter containing 2101 words. * *p* < .05, ** *p* < .01, *** *p* < .001. Abbreviations: FFD, first fixation duration; GD, gaze duration; FPF, first-pass reading fixation proportion.
