# Supplementary material for: The database of eye-movement measures on words in Chinese reading
Source: Sci Data. 2022 Jul 15;9:411. doi: 10.1038/s41597-022-01464-6 (PMC9287311; doi:10.1038/s41597-022-01464-6)
Supplement: Supplementary file 2 — Supplementary Table 2 [file 41597_2022_1464_MOESM2_ESM.docx]

**Supplementary Table 2**

*Results for the Effects of Word Frequency and Word Length on the Main Eye-Movement Measures in Four Quarters of Words Divided Based on the Number of Observations*

| Dependent  variables | Independent variables | Quarters | *b* value | Cohen’s *d* | *t* value |
| --- | --- | --- | --- | --- | --- |
| FFD | Log-transformed word frequency | Quarter 1 | -9.677 | -0.141 | -4.478^***^ |
|  |  | Quarter 2 | -9.140 | -0.174 | -5.713^***^ |
|  |  | Quarter 3 | -6.044 | -0.152 | -5.283^***^ |
|  |  | Quarter 4 | -4.212 | -0.187 | -6.495^***^ |
|  | 2-char words vs 1-char words | Quarter 1 | -3.018 | -0.067 | -0.815 |
|  |  | Quarter 2 | -10.143 | -0.302 | -3.436^***^ |
|  |  | Quarter 3 | -8.021 | -0.306 | -3.407^***^ |
|  |  | Quarter 4 | -7.724 | -0.440 | -6.212^***^ |
|  | 3-char words vs 2-char words | Quarter 1 | -17.474 | -0.387 | -5.245^***^ |
|  |  | Quarter 2 | -4.294 | -0.128 | -1.480 |
|  |  | Quarter 3 | -12.498 | -0.476 | -5.209^***^ |
|  |  | Quarter 4 | -7.066 | -0.403 | -2.963^**^ |
|  | 4-char words vs 3-char words | Quarter 1 | -1.762 | -0.039 | -0.383 |
|  |  | Quarter 2 | -16.468 | -0.490 | -4.751^***^ |
|  |  | Quarter 3 | -2.665 | -0.102 | -0.738 |
|  |  | Quarter 4 | -3.163 | -0.180 | -0.767 |
| GD | Log-transformed word frequency | Quarter 1 | -33.019 | -0.231 | -8.239^***^ |
|  |  | Quarter 2 | -25.612 | -0.214 | -7.997^***^ |
|  |  | Quarter 3 | -18.340 | -0.214 | -8.107^***^ |
|  |  | Quarter 4 | -8.167 | -0.189 | -7.507^***^ |
|  | 2-char words vs 1-char words | Quarter 1 | 13.321 | 0.142 | 1.939 |
|  |  | Quarter 2 | 11.602 | 0.151 | 1.964^*^ |
|  |  | Quarter 3 | 12.650 | 0.223 | 2.718^**^ |
|  |  | Quarter 4 | 13.260 | 0.395 | 6.357^***^ |
|  | 3-char words vs 2-char words | Quarter 1 | 29.093 | 0.309 | 4.709^***^ |
|  |  | Quarter 2 | 68.238 | 0.891 | 11.753^***^ |
|  |  | Quarter 3 | 35.386 | 0.624 | 7.460^***^ |
|  |  | Quarter 4 | 44.548 | 1.328 | 11.135^***^ |
|  | 4-char words vs 3-char words | Quarter 1 | 80.517 | 0.856 | 9.443^***^ |
|  |  | Quarter 2 | 4.290 | 0.056 | 0.618 |
|  |  | Quarter 3 | 37.099 | 0.655 | 5.193^***^ |
|  |  | Quarter 4 | 33.864 | 1.010 | 4.891^***^ |
| FPF | Log-transformed word frequency | Quarter 1 | -0.074 | -0.237 | -10.426^***^ |
|  |  | Quarter 2 | -0.041 | -0.151 | -7.387^***^ |
|  |  | Quarter 3 | -0.020 | -0.081 | -4.350^***^ |
|  |  | Quarter 4 | -0.014 | -0.066 | -4.781^***^ |
|  | 2-char words vs 1-char words | Quarter 1 | 0.230 | 1.173 | 18.218^***^ |
|  |  | Quarter 2 | 0.282 | 1.795 | 25.567^***^ |
|  |  | Quarter 3 | 0.292 | 1.831 | 33.709^***^ |
|  |  | Quarter 4 | 0.294 | 1.858 | 58.763^***^ |
|  | 3-char words vs 2-char words | Quarter 1 | 0.096 | 0.488 | 10.029^***^ |
|  |  | Quarter 2 | 0.112 | 0.710 | 9.532^***^ |
|  |  | Quarter 3 | 0.131 | 0.821 | 13.300^***^ |
|  |  | Quarter 4 | 0.151 | 0.957 | 12.147^***^ |
|  | 4-char words vs 3-char words | Quarter 1 | 0.023 | 0.117 | 1.682 |
|  |  | Quarter 2 | 0.016 | 0.102 | 1.197 |
|  |  | Quarter 3 | 0.031 | 0.196 | 2.036^*^ |
|  |  | Quarter 4 | 0.043 | 0.273 | 2.016^*^ |

*Note.* Quarters of each measure were divided based on the number of observations of words in ascending order, with each quarter containing 2101 words. * *p* < .05, ** *p* < .01, *** *p* < .001. Abbreviations: FFD, first fixation duration; GD, gaze duration; FPF, first-pass reading fixation proportion.
